# Supplementary material for: Effects of Different Farming Models on Muscle Quality, Intestinal Microbiota Diversity, and Liver Metabolism of Rice Field Eel (Monopterus albus)
Source: Foods. 2025 Jul 5;14(13):2383. doi: 10.3390/foods14132383 (PMC12248573; doi:10.3390/foods14132383)
Supplement: Supplementary file 1 [file foods-14-02383-s001.zip › foods-3686019-supplementary.pdf]

Supplementary Materials

**Table S1.** Significantly differentially abundant metabolites in the liver of *M. albus*: CG *vs.* RG

| ID | Metabolite | p-Value | VIP | regulated | Metabolic pathway |
|---|---|---|---|---|---|
| MN22469.neg | FAD | < 0.001 | 2.12 | up | Biosynthesis of cofactors; Riboflavin metabolism |
| MP18732.pos | Flavinmononucleotide | > 0.001 | 1.98 | up | Biosynthesis of cofactors; Riboflavin metabolism |
| MN3498.neg | Phosphoenolpyruvic acid | 0.017 | 1.81 | up | Biosynthesis of cofactors; Biosynthesis of amino acids; Carbon metabolism |
| MN2003.neg | L-Cysteine | > 0.001 | 2.43 | up | Biosynthesis of cofactors; Biosynthesis of amino acids; Carbon metabolism; D-Amino acid metabolism; Pantothenate and CoA biosynthesis |
| MP8180.pos | Biotin | 0.002 | 1.85 | up | Biosynthesis of cofactors |
| MP15480.pos | RIBOFLAVIN | 0.020 | 1.63 | up | Biosynthesis of cofactors; Riboflavin metabolism |
| MP9514.pos | Vitamin A | 0.030 | 1.63 | up | Biosynthesis of cofactors |
| MN7900.neg | D-4'-Phosphopantothenate | 0.013 | 1.93 | up | Biosynthesis of cofactors; Pantothenate and CoA biosynthesis |
| MN4165.neg | L-glutamic | 0.011 | 1.86 | up | Biosynthesis of amino acids; D-Amino acid metabolism |
| MN3746.neg | 3-Isopropylmalic acid | > 0.001 | 2.26 | up | Biosynthesis of amino acids |
| MP32759.pos | PC(15:0) | 0.049 | 1.53 | up | Arachidonic acid metabolism |
| MN9890.neg | 20-hydroxy Leukotriene B4 | 0.040 | 1.77 | up | Arachidonic acid metabolism |
| MN10483.neg | Prostaglandin G2 | 0.038 | 1.77 | up | Arachidonic acid metabolism |
| MP11423.pos | 5,6-epoxy-8Z,11Z,14Z-eicos atrienoic acid | 0.015 | 1.80 | up | Arachidonic acid metabolism |
| MN8733.neg | 16-HETE | 0.015 | 1.96 | up | Arachidonic acid metabolism |
| MN1517.neg | (2R)-2,3-Dihydroxypropan oic acid | > 0.001 | 2.25 | up | Carbon metabolism |
| MP23842.pos | LysoPC(20:4) | > 0.001 | 2.09 | up | Glycerophospholipid metabolism |
| MP22047.pos | LysoPC(18:1) | 0.007 | 1.86 | up | Glycerophospholipid metabolism |
| MP24039.pos | LysoPC(20:2) | > 0.001 | 2.06 | up | Glycerophospholipid metabolism |
| MP25118.pos | LysoPC(22:4) | > 0.001 | 2.10 | up | Glycerophospholipid metabolism |
| MN9118.neg | dIMP | 0.038 | 1.76 | up | Purine metabolism |
| MN9341.neg | Imidazoleacetic acid ribotide | 0.006 | 2.15 | up | Histidine metabolism |
| MN8791.neg | D-Pantothenoyl-L-cysteine | 0.014 | 2.02 | up | Biosynthesis of cofactors; Biosynthesis of amino acids; Carbon metabolism; D-Amino acid metabolism |
| MN2362.neg | Oxaloacetic acid | 0.027 | 1.71 | down | Biosynthesis of cofactors |
| MP11705.pos | Glutathione | 0.006 | 1.91 | down | Biosynthesis of cofactors; Purine metabolism |
| MN9715.neg | Inosinic acid | 0.031 | 1.76 | down | Biosynthesis of cofactors; Pantothenate and CoA biosynthesis |
| MN2940.neg | pantoic acid | 0.044 | 1.74 | down | Biosynthesis of cofactors; Biosynthesis of amino acids; Carbon metabolism; D-Amino acid metabolism |
| MN3549.neg | Dihydroxyacetone phosphate | 0.006 | 1.94 | down | Biosynthesis of amino acids; Carbon metabolism |

**Table S1(continued).** Significantly differentially abundant metabolites in the liver of *M. albus*: CG *vs.* RG

| ID | Metabolite | p-Value | VIP | regulated | Metabolic pathway |
|---|---|---|---|---|---|
| MP2660.pos | L-Proline | 0.027 | 1.64 | down | Biosynthesis of amino acids; D-Amino acid metabolism |
| MN2370.neg | L-Asparagine | 0.037 | 1.75 | down | Biosynthesis of amino acids |
| MN10291.neg | Guanosine 5'-monophosphate | 0.029 | 1.78 | down | Purine metabolism |
| MN13272.neg | Adenosine 5'-phosphosulfate | 0.034 | 1.69 | down | Purine metabolism |
| MP4183.pos | Acetylhistamine | 0.039 | 1.44 | down | Histidine metabolism |
| MP5985.pos | L-Histidine trimethylbetaine | 0.041 | 1.43 | down | Histidine metabolism |
| MP3693.pos | Imidazolepropionic acid | 0.011 | 1.74 | down | Histidine metabolism |

**Table S2.** Significantly differentially abundant metabolites in the liver of *M. albus*: RAG *vs.* RG

| ID | Metabolite | p-Value | VIP | regulated | Metabolic pathway |
|---|---|---|---|---|---|
| MP11705.pos | Glutathione | > 0.001 | 2.61 | up | Biosynthesis of cofactors; Glutathione metabolism |
| MN2003.neg | L-Cysteine | > 0.001 | 2.91 | up | Biosynthesis of cofactors; Biosynthesis of amino acids; Pantothenate and CoA biosynthesis; Glutathione metabolism; D-Amino acid metabolism |
| MP7712.pos | L-Valine | 0.048 | 1.66 | up | Biosynthesis of cofactors; Biosynthesis of amino acids; Pantothenate and CoA biosynthesis |
| MN6552.neg | MANNOSE 6-PHOSPHATE | 0.049 | 1.97 | up | Biosynthesis of cofactors |
| MN4547.neg | D-Erythrose 4-phosphate | 0.017 | 2.36 | up | Biosynthesis of cofactors; Biosynthesis of amino acids; |
| MP6221.pos | Spermine | 0.011 | 2.13 | up | Biosynthesis of cofactors; Arginine and proline metabolism; Pantothenate and CoA biosynthesis; Glutathione metabolism; beta-Alanine metabolism |
| MP7314.pos | Porphobilinogen | 0.019 | 2.09 | up | Biosynthesis of cofactors |
| MN3549.neg | Dihydroxyacetone phosphate | 0.012 | 2.33 | up | Biosynthesis of amino acids; Glycerophospholipid metabolism; Glycolysis / Gluconeogenesis |
| MP2660.pos | L-Proline | 0.222 | 2.00 | up | Biosynthesis of amino acids; Arginine and proline metabolism; D-Amino acid metabolism |
| MN6851.neg | N-Acetyl-L-glutamyl 5-phosphate | 0.049 | 2.05 | up | Biosynthesis of amino acids |
| MP2589.pos | Creatinine | > 0.001 | 2.42 | up | Arginine and proline metabolism |
| MP3338.pos | 4-HYDROXY PROLINE | 0.047 | 1.67 | up | Arginine and proline metabolism; D-Amino acid metabolism |
| MP22047.pos | LysoPC(18:1) | 0.024 | 1.92 | up | Glycerophospholipid metabolism |
| MP33124.pos | LysoPC(14:1) | 0.041 | 1.89 | up | Glycerophospholipid metabolism |
| MP23937.pos | LysoPC(20:5) | 0.021 | 1.42 | up | Glycerophospholipid metabolism |
| MP2165.pos | Malonic acid | 0.040 | 1.81 | up | beta-Alanine metabolism |
| MP5726.pos | L-Kynurenine | 0.029 | 1.90 | down | Biosynthesis of cofactors |
| MN10012.neg | 2,5-diamino-6-(5-phospho-D-ribitylamino)pyrimidin-4(3H)-one | 0.031 | 2.17 | down | Biosynthesis of cofactors |
| MP8064.pos | LUMICHROME | 0.016 | 2.11 | down | Riboflavin metabolism |
| MN3873.neg | D-(+)-Glucose | 0.022 | 2.31 | down | Glycolysis / Gluconeogenesis |

**Table S3.** Significantly differentially abundant metabolites in the liver of *M. albus*: CG *vs.* RAG

| ID | Metabolite | p-Value | VIP | regulated | Metabolic pathway |
|---|---|---|---|---|---|
| MN22469.neg | FAD | < 0.001 | 2.21 | up | Biosynthesis of cofactors; Riboflavin metabolism |
| MP18732.pos | Flavin mononucleotide | > 0.001 | 2.07 | up | Biosynthesis of cofactors; Riboflavin metabolism |
| MN349 8.neg | Phosphoenolpyruvic acid | 0.006 | 1.90 | up | Biosynthesis of cofactors; Biosynthesis of amino acids; Carbon metabolism |
| MP15480.pos | RIBOFLAVIN | 0.003 | 1.90 | up | Biosynthesis of cofactors; Riboflavin metabolism |
| MN7900.neg | D-4'-Phosphopantothenate | 0.007 | 1.88 | up | Biosynthesis of cofactors; Pantothenate and CoA biosynthesis |
| MN10012.neg | 2,5-diamino-6-(5-phospho-D-ribitylamino)pyrimidin-4(3H)-one | 0.048 | 1.66 | up | Biosynthesis of cofactors; Riboflavin metabolism |
| MP3951.pos | L-Lysine | 0.033 | 1.70 | up | Biosynthesis of amino acids |
| MN1517.neg | (2R)-2,3-Dihydroxypropanoic acid | 0.004 | 1.94 | up | Carbon metabolism; Glycine, serine and threonine metabolism |
| MP10223.pos | Ceramide (d18:1/16:0) | 0.040 | 1.64 | up | Sphingolipid metabolism |
| MP11258.pos | Sphingosine | 0.040 | 1.63 | up | Sphingolipid metabolism |
| MN2672.neg | Ethanolamine phosphate | 0.020 | 1.78 | up | Sphingolipid metabolism |
| MP19915.pos | Psychosine | 0.008 | 1.86 | up | Sphingolipid metabolism |
| MP12288.pos | Phytosphingosine | < 0.001 | 2.13 | up | Sphingolipid metabolism |
| MP23842.pos | LysoPC(20:4) | 0.005 | 2.02 | up | Glycerophospholipid metabolism |
| MP22047.pos | LysoPC(18:1) | < 0.001 | 2.12 | up | Glycerophospholipid metabolism |
| MP24039.pos | LysoPC(20:2) | < 0.001 | 2.21 | up | Glycerophospholipid metabolism |
| MP21459.pos | LysoPC(16:1) | 0.005 | 1.88 | up | Glycerophospholipid metabolism |
| MP25118.pos | LysoPC(22:4) | 0.002 | 2.06 | up | Glycerophospholipid metabolism |
| MP14097.pos | Dehydroepiandrosterone sulfate | < 0.001 | 2.31 | up | Steroid hormone biosynthesis |
| MP14112.pos | Tetrahydrocorticosterone | 0.024 | 1.64 | up | Steroid hormone biosynthesis |
| MP13886.pos | CORTEXOLONE | 0.029 | 1.64 | up | Steroid hormone biosynthesis |
| MN11465.neg | 3beta-Hydroxypregn-5-en-20-one sulfate | 0.013 | 1.77 | up | Steroid hormone biosynthesis |
| MP4132.pos | Xanthine | 0.034 | 1.74 | up | Purine metabolism |
| MP8064.pos | LUMICHROME | 0.017 | 1.74 | up | Riboflavin metabolism |
| MP3341.pos | Creatine | > 0.001 | 2.03 | up | Glycine, serine and threonine metabolism |
| MN22260.neg | Phosphatidylserine | 0.017 | 1.72 | up | Glycine, serine and threonine metabolism |
| MN8791.neg | D-Pantothenoyl-L-cysteine | 0.009 | 1.83 | up | Pantothenate and CoA biosynthesis |
| MN9683.neg | Adenosine 5'-monophosphate | 0.011 | 1.90 | down | Biosynthesis of cofactors; Purine metabolism |
| MN2362.neg | Oxaloacetic acid | 0.039 | 1.62 | down | Biosynthesis of cofactors; Biosynthesis of amino aci ds; Carbon metabolism |
| MP11705.pos | Glutathione | < 0.001 | 2.33 | down | Biosynthesis of cofactors; |

**Table S3(continued).** Significantly differentially abundant metabolites in the liver of *M. albus*: CG *vs.* RAG

| ID | Metabolite | p-Value | VIP | regulated | Metabolic pathway |
|---|---|---|---|---|---|
| MP2220.pos | L-Serine | 0.037 | 1.63 | down | Biosynthesis of cofactors; Biosynthesis of amino acids; Carbon metabolism; Sphingolipid metabolism; Glycerophospholipid metabolism; Glycine, serine and threonine metabolism |
| MN8851.neg | URIDINE-5-MONOPHOSPHATE | 0.040 | 1.60 | down | Biosynthesis of cofactors |
| MN18387.neg | Oxidized glutathione | 0.021 | 1.80 | down | Biosynthesis of cofactors |
| MN9715.neg | Inosinic acid | 0.020 | 1.84 | down | Biosynthesis of cofactors; Purine metabolism |
| MP7712.pos | L-Valine | 0.007 | 1.88 | down | Biosynthesis of cofactors; Biosynthesis of amino acids; Pantothenate and CoA biosynthesis |
| MN6552.neg | MANNOSE 6-PHOSPHATE | 0.020 | 1.83 | down | Biosynthesis of cofactors |
| MP6221.pos | Spermine | 0.012 | 1.78 | down | Biosynthesis of cofactors; Pantothenate and CoA biosynthesis |
| MP7314.pos | Porphobilinogen | 0.016 | 1.88 | down | Biosynthesis of cofactors |
| MN3549.neg | Dihydroxyacetone phosphate | 0.003 | 2.10 | down | Biosynthesis of amino acids; Carbon metabolism |
| MP2660.pos | L-Proline | 0.002 | 1.99 | down | Biosynthesis of amino acids |
| MN2370.neg | L-Asparagine | 0.002 | 2.15 | down | Biosynthesis of amino acids |
| MN13762.neg | Xylulose 5-phosphate | 0.039 | 1.69 | down | Biosynthesis of amino acids; Carbon metabolism |
| MN7580.neg | D-Sedoheptulose 7-phosphate | < 0.001 | 2.09 | down | Biosynthesis of amino acids; Carbon metabolism |
| MN6899.neg | Estrone | 0.006 | 1.88 | down | Steroid hormone biosynthesis |
| MN6957.neg | -Estradiol | 0.039 | 1.60 | down | Steroid hormone biosynthesis |
| MN10291.neg | Guanosine 5'-monophosphate | 0.048 | 1.64 | down | Purine metabolism |
| MP9439.pos | Adenosine | <0.001 | 2.08 | down | Purine metabolism |
| MN13272.neg | Adenosine 5'-phosphosulfate | 0.015 | 1.92 | down | Purine metabolism |
| MP2746.pos | BETAINE | 0.032 | 1.56 | down | Glycine, serine and threonine metabolism |
